# IFN-*α*-2b Reduces Postoperative Arthrofibrosis in Rats by Inhibiting Fibroblast Proliferation and Migration through STAT1/p21 Signaling Pathway

**DOI:** 10.1155/2023/1699946

**Published:** 2023-03-04

**Authors:** Zhendong Liu, Zhehao Fan, Rui Wang, Xiaolei Li, Hui Chen, Jingcheng Wang

**Affiliations:** ^1^Clinical Medical College of Yangzhou University, Subei People's Hospital of Jiangsu Province, Yangzhou 225001, China; ^2^Qilu Hospital of Shandong University Dezhou Hospital, Dezhou 253000, China

## Abstract

**Objective:**

To investigate the effect of IFN-*α*-2b in preventing postoperative arthrofibrosis in rats, its antiproliferation effect on fibroblasts in vitro, and its molecular mechanism.

**Methods:**

The rat model of arthrofibrosis was established and treated with different concentrations of drugs. Knee specimens were collected for histological and immunohistochemical staining to observe the effect of IFN-*α*-2b on arthrofibrosis in rats. The biological information was further mined according to the database data, and the possible regulatory mechanism of IFN-*α*-2b on fibroblasts was analyzed. The inhibitory effect of IFN-*α*-2b on fibroblast proliferation and migration in vitro was detected by cell counting kit-8 (CCK-8), immunofluorescence analysis, cell cycle test, EdU assay, wound healing test, and Transwell method, and the analysis results were verified by Western blotting method.

**Results:**

The test results of rat knee joint specimens showed that IFN-*α*-2b significantly inhibited the degree of fibrosis after knee joint surgery, the number of fibroblasts in the operation area was less than that of the control group, and the expression of collagen and proliferation-related proteins decreased. In vitro experimental results show that IFN-*α*-2b can inhibit the proliferation and migration of fibroblasts. According to the results of database analysis, it is suggested that the STAT1/P21 pathway may be involved, and it has been verified and confirmed by Western blotting and other related methods.

**Conclusion:**

IFN-*α*-2b can reduce surgery-induced arthrofibrosis by inhibiting fibroblast proliferation and migration, which may be related to the regulation of STAT1/p21 signaling pathway.

## 1. Introduction

Arthrofibrosis is a common complication in joints after trauma and surgery, which is characterized by the production of excessive fibrous scar tissue in joints [1–3]. Previous studies have shown that arthrofibrosis is related to the excessive proliferation of fibroblasts in the surgical area. The excessive proliferated fibroblasts will migrate to the area after surgery, secreting excessive extracellular matrix (ECM) and collagen deposition, which eventually leads to arthrofibrosis [4, 5]. As the overhyperplasia of fibrosis tissue causes pain and limits the normal range of joint activity, it can seriously affect the patient's postoperative life [6, 7].

In recent years, many strategies have been adopted, such as all-round movements of the knee joint after anesthesia, arthroscopic cleaning of joint cavity, and local drug treatment to reduce the formation of postoperative arthrofibrosis [8–10]. Other studies have reported that the best treatment for arthrofibrosis is early identification and intervention [11]. Therefore, the early application of drugs to prevent arthrofibrosis deserves attention. But, although some achievements have been made in the research of locally applied drugs, there are still considerable limitations before clinical trials due to the side effects of the above drugs or the effect of the route of administration.

IFN-*α*-2b is a kind of cytokine with many biological activities such as antiviral, antitumor, antiproliferation, and immunomodulation. It has good drug tolerance in the body, even in high-dose application [12]. Previous studies have shown that IFN-*α*-2b can treat some fibroproliferative diseases (such as hypertrophic scar and keloid) [13]. In addition, it also plays an active preventive role in scar formation after glaucoma filtration surgery and scar formation after cleft palate surgery [14–16]. It can not only inhibit the proliferation of fibroblasts but also reduce the formation of collagen, thus preventing the formation of fibrosis. However, there is no research on IFN-*α*-2b for arthrofibrosis in the existing literature. Based on the above research background, we choose IFN-*α*-2b on rats for the prevention of postoperative arthrofibrosis and the treatment of fibroblasts in vitro in order to explore its effect of preventing knee arthrofibrosis and the possible mechanism involved.

## 2. Materials and Methods

### 2.1. Reagent

The reagent used is recombinant human interferon alpha 2b (IFN-*α*-2b), purity > 95%, originated from GenScript Biotechnology Co., Ltd. (Nanjing, China).

### 2.2. Animals

The animal research in this experiment was approved by the Animal Research Committee of Yangzhou University. All rats received strict care. A total of 36 SD male rats weighing about 300 g were selected and randomly divided into 3 groups (12 in each group).

### 2.3. Establishment of Arthrofibrosis Model

After the rats were successfully anesthetized, the knee joint was opened through the medial approach of the patella, and the medial and lateral femoral condyles were fully exposed. The cortical bone of about 4^∗^4 mm was excised until the cancellous bone was exposed, and the articular cartilage was intact; then, the wound was covered with saline or IFN-*α*-2b gauze for 10 minutes. After hemostasis, the gauze was removed to suture the joint capsule and skin. After the operation, antibiotics were applied for 3 consecutive days (intramuscularly, 50 mg/kg), and saline or corresponding concentration of IFN-*α*-2b 50 *μ*l was injected locally in the operation knee joint 3 times a week [17].

### 2.4. Histological Analysis

The experiment ended 4 weeks after surgery. Six rats were randomly selected from each group, and knee joint specimens were collected for histological analysis. The knee joint specimens were fixed with 4% paraformaldehyde for 48 hours, fully decalcified in ethylenediamine tetra acetic acid (EDTA), and embedded in paraffin. 4 *μ*m serial sections were deparaffinized to water, and hematoxylin-eosin (HE) staining was performed to detect the degree of fibrosis and the number of fibroblasts, and Masson's trichrome staining was used to observe the collagen production. The collagen was stained with picric acid-Sirius red, and the type I collagen and type III collagen were observed and distinguished under a polarized light microscope; the optical density value of the stained image and the cell count analysis was detected by ImageJ software.

### 2.5. Immunohistochemical Staining

The sections were deparaffinized and rehydrated. Sodium citrate buffer was used to activate antigenicity, 3% H_2_O_2_ inhibited peroxidase activity, and the sections were washed with PBS solution for 3 time. Then, the primary antibodies (anti-PCNA, anti-collagen I, and anti-*α*-SMA) were incubated overnight at 4°C. The sections were incubated with anti-mouse IgG for 30 minutes at room temperature, and the DAB kit was used to detect antibody binding. Finally, hematoxylin counterstaining was carried out, and the results were observed and photographed under an optical microscope.

### 2.6. Dataset Selection and DEG Identification

The gene expression dataset GSE38652 was obtained from the GEO database (https://www.ncbi.nlm.nih.gov/geo/). The screening basis included (a) IFN-*α*-2b as the processing factor, (b) fibroblasts as the intervention object, and (c) the organism as Homo sapiens. GSE38652 is based on the GPL10558 (Illumina HumanHT-12 V4.0 expression beadchip) platform, and all data can be obtained online for free. Use GEO2R (https://www.ncbi.nlm.nih.gov/geo/geo2r/) online software to analyze raw data and identify differentially expressed genes (DEGs). *P* < 0.05 and |logFC| ≥ 1 were used as cut-off criteria to obtain DEGs.

### 2.7. Enrichment Analyses of DEGs and PPI Network

The protein-protein interaction (PPI) network was established by STRING database (http://string-db.org) [18]. In this research, the interaction with high confidence > 0.7 was statistically significant. In order to explore more biological information related to DEGs and obtain more comprehensive gene and protein function, we conducted Gene Ontology (GO) enrichment analysis and Kyoto Encyclopedia of Genes and Genomes (KEGG) pathway enrichment analysis through Metascape (http://metascape.org) [19]. GO enrichment analysis includes biological processes (BP), cell component (CC), and molecular function (MF). In addition, we also performed TRRUST transcriptional regulatory network analysis [20].

### 2.8. Fibroblast Culture and IFN-*α*-2b Treatments

The human fibroblast cell line was offered by Jenino Biotech Co., Ltd. (Guangzhou, China) and then cultured in a medium containing 15% fetal bovine serum (FBS; Gemini, USA) and 1% streptomycin/penicillin (Beyotime, Shanghai, China), at 37°C with 5% CO_2_. Select fibroblasts between 3 and 5 generations for subsequent experiments. Fibroblasts were treated with IFN-*α*-2b in four concentration groups (0, 1000, 5000, and 10000 IU/ml) [17]. In the mechanism research group, we pretreated fibroblasts with 50 *μ*M fludarabine (a specific Stat1 inhibitor) for 2 hours and then changed to a medium containing different concentrations of IFN-*α*-2b to continue incubating [21].

### 2.9. Cell Viability Assay

Firstly, 100 *μ*l cell suspension was planted in 96-well culture plate. When the cell density reached 60%-70%, different concentrations of IFN-*α*-2b were added to treat fibroblasts. After drug stimulation, 10 *μ*l CCK-8 (Dojindo, Tokyo, Japan) solution was added and then cultured at 37°C for 2 hours. Finally, the absorbance at 450 nm was measured.

### 2.10. Cell Cycle Analysis

Fibroblasts were treated with IFN-*α*-2b of 5,000 U/ml for 48 hours and then operated according to the instructions of Cell Cycle Testing Kit (Beyotime, Shanghai, China). Cells were collected, centrifuged at 2,000 r/min for 5 minutes, washed with precooled PBS, fixed with 70% absolute ethanol, and fixed overnight at 4°C. After centrifugation again, cells were resuspended with 500 *μ*l propidium iodide staining solution prepared in advance, incubated at room temperature for 30 minutes in the dark, and then, flow cytometry was performed. Finally, the distribution of cells in different periods was calculated by ModFit LT software.

### 2.11. Cell Immunofluorescence Imaging Analysis

Fibroblasts were fixed in 4% paraformaldehyde for 20 minutes, infiltrated with 0.5% Triton X-100 for 10 minutes, and blocked with 5% goat serum at room temperature for 30 minutes. Then, the primary antibody mixture (type I collagen and *α*-SMA) was dripped onto the cell slide and incubated overnight at 4°C. After PBST cleaning, the secondary antibody was incubated in the dark at room temperature for 1 hour. After cleaning, DAPI was stained in the dark for 10 minutes. Finally, the images were collected under the fluorescence microscope (Zeiss, Germany).

### 2.12. EdU Incorporation Assay

Cell-Light KFluor555 EdU Kit (KeyGEN, Nanjing, China) was used to detect the proliferation of fibroblasts. Operate according to the instructions. First, plant fibroblasts on glass cell slides in a 6-well plate at a cell density of 1 × 10^5^/well. The cells were cultured at 37°C overnight, and different concentrations of interferon-*α*-2b were added to the culture medium for 48 hours. After supplementing with 10  *μ*M EdU and incubating for 2 h, the cells were fixed with 4% paraformaldehyde for 15 minutes, and then infiltrate with 0.5% Triton X-100 for 20 minutes. Finally, stain with Hoechst 33342, incubate in the dark at room temperature for 10 min, and observe the positive staining under an upright fluorescence microscope.

### 2.13. Cell Migration Assay

Wound healing test and Transwell migration test were used to evaluate cell migration behavior. In short, put a sterilized culture-inserts in the center of each hole of the 12 well culture plate, add 70 ml of cell suspension in each interval of the culture-inserts, place it in the cell incubator until the cells are completely fused at the bottom, and then gently remove the culture-inserts. Wash with PBS for 3 times, and then, serum-free medium and detection reagents were added; observe and collect images at different time points (0, 12, 24, and 48 h).

Polycarbonate membrane Transwell filter was selected for Transwell assay (Corning Company, New York, USA). Firstly, 600 *μ*l complete culture medium was added into each well of the 24-well plate, and then, 100 *μ*l of serum-free fibroblast suspension (5 × 10^4^ cells) was transferred to the upper layer of the Transwell chamber and treated with different reagents. After cultured in the cell incubator for 24 hours, the chamber was lightly washed with PBS, fixed with 4% paraformaldehyde, stained with crystal violet, and gently wiped the upper cells of the chamber with a cotton swab. Finally, the images were collected under Zeiss inverted microscope. The wound healing rate and migrating transmembrane cells were calculated by ImageJ software.

### 2.14. Western Blotting Analysis

According to the instructions of RIPA Lysis Solution (Beyotime, Shanghai, China), the total proteins of different groups of fibroblasts were extracted. The same amount of total protein (60 *μ*g/lane) was electrophoresed on 10% or 12% SDS-PAGE and then transferred to polyvinylidene difluoride membranes (Millipore, Bedford, MA) at low temperature. Block with 5% skimmed milk or 3% bovine serum albumin (BSA) at room temperature for 2 hours, incubate the primary antibody overnight at 4°C, and then incubate the secondary antibody at room temperature for 2 hours. Finally, the enhanced chemiluminescence detection kit (ECL Plus kit, Beyotime) was used to detect protein bands.

The mouse monoclonal antibodies PCNA (#2586), rabbit monoclonal antibodies cyclin A (#67955), horseradish peroxidase-conjugated goat anti-mouse (#7056), and goat anti-rabbit (#7074) antibodies were purchased from Cell Signaling Technology (Beverly, MA, USA). The rabbit monoclonal antibodies STAT1 (ab109320) and phospho-STAT1 (ab109461) were offered by Abcam (Cambridge, UK). The mouse monoclonal *α*-SMA (67735-1-Ig), rabbit polyclonal antibody P21 (10355-1-AP), collagen type I (14695-1-AP), *β*-actin (20536-1-AP), and GAPDH (10494-1-AP) were offered by Proteintech Group (Wuhan, China).

### 2.15. Statistical Analysis

All data in this study were analyzed by SPSS 19.0 statistical software. The data were expressed as mean ± standard deviation. Data were analyzed by one-way analysis of variance (ANOVA), followed by Tukey's test for comparison between groups. *P* < 0.05 was considered statistically significant.

## 3. Results

### 3.1. IFN-*α*-2b Improves Postoperative Arthrofibrosis in Rats

**Figure 1:**
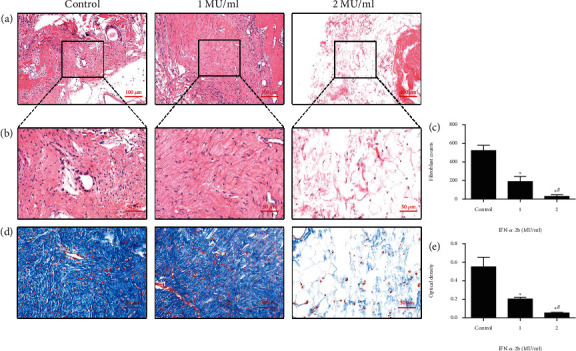
Histological evaluation of joint fibrosis in different groups. (a, b) HE staining showed fibroblasts in each group of joint fibrotic tissue; (c) the number of fibroblasts decreased with the increase of IFN-*α*-2b concentration; (d) Masson's staining showed the content of collagen in knee tissues of each group. (e) The results of optical density analysis suggest that IFN-*α*-2b can effectively reduce the production of collagen. ^∗^Compared with the control group. ^#^Comparison between the two groups of IFN-*α*-2b medications, *P* < 0.05 (*n* = 6).

According to the HE staining results, the number of scar fibroblasts and dense fibrous tissue around the knee operation area in the IFN-*α*-2b group decreased, the tissue structure was sparse, and the degree of fibrosis was improved (Figures 1(a) and 1(b)); the number of fibroblasts decreased with the increase of IFN-*α*-2b concentration (Figure 1(c)). Masson's staining was used to evaluate the degree of collagen synthesis in arthrofibrosis after local administration of IFN-*α*-2b. The staining results showed that collagen synthesis in arthrofibrosis decreased after application of IFN-*α*-2b, especially in the high-dose application group (Figure 1(d)); the results of optical density analysis also suggest that IFN-*α*-2b can effectively reduce the production of collagen (Figure 1(e)).

**Figure 2:**
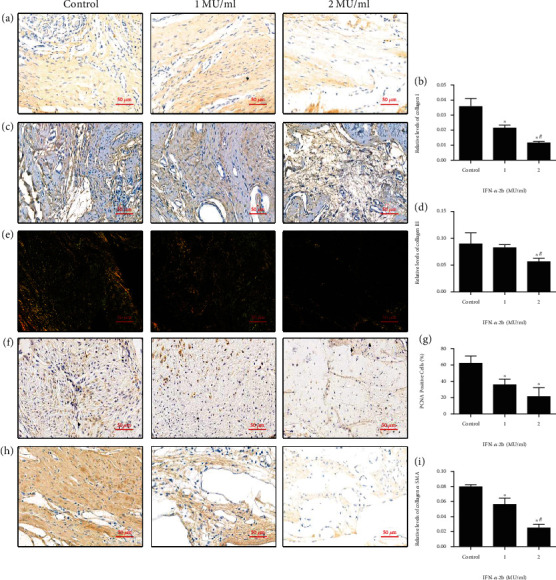
Immunohistochemical staining images of joint fibrosis in different groups (×400 magnification). (a, c) The results of immunohistochemical staining of type I collagen and type III collagen in joint fibrotic tissue. (b, d) The positive expression of collagen immunohistochemical staining in the IFN-*α*-2b group was significantly reduced. IFN-*α*-2b effectively inhibits the formation of collagen I and collagen III in fibrotic tissues. (e) The result of Sirius red staining was visualized by polarized light microscope, in which type I collagen was yellow and type III collagen was green. The image shows that collagen fibers decrease with increasing drug concentration. (f, h) Immunohistochemical staining image of PCNA and *α*-SMA. (g, i) The IFN-*α*-2b medication group can significantly reduce the expression of PCNA and *α*-SMA. ^∗^Compared with the control group. ^#^Comparison between the two groups of IFN-*α*-2b medications, *P* < 0.05 (*n* = 6).

Immunohistochemical staining showed that the expression of type I collagen in the IFN-*α*-2b treatment group was lower than that in the normal saline application group (Figures 2(a) and 2(b)). The same was true for the expression of type III collagen (Figures 2(c) and 2(d)). Sirius red staining showed that type I collagen fibers (red or yellow) and type III collagen fibers (green) decreased in the IFN-*α*-2b treatment group (Figure 2(e)). Similarly, immunohistochemical staining of proliferation- and migration-related proteins PCNA (Figure 2(f)) and *α*-SMA (Figure 2(h)) showed that the expression of the IFN-*α*-2b-treated group was significantly lower than that of the control group (Figures 2(g) and 2(i)). In conclusion, the above in vivo experimental results suggest that IFN-*α*-2b has the potential to improve arthrofibrosis in rats.

### 3.2. DEG Acquisition and PPI Network Relationship, GO, KEGG, and TRRUST Enrichment Analyses

**Figure 3:**
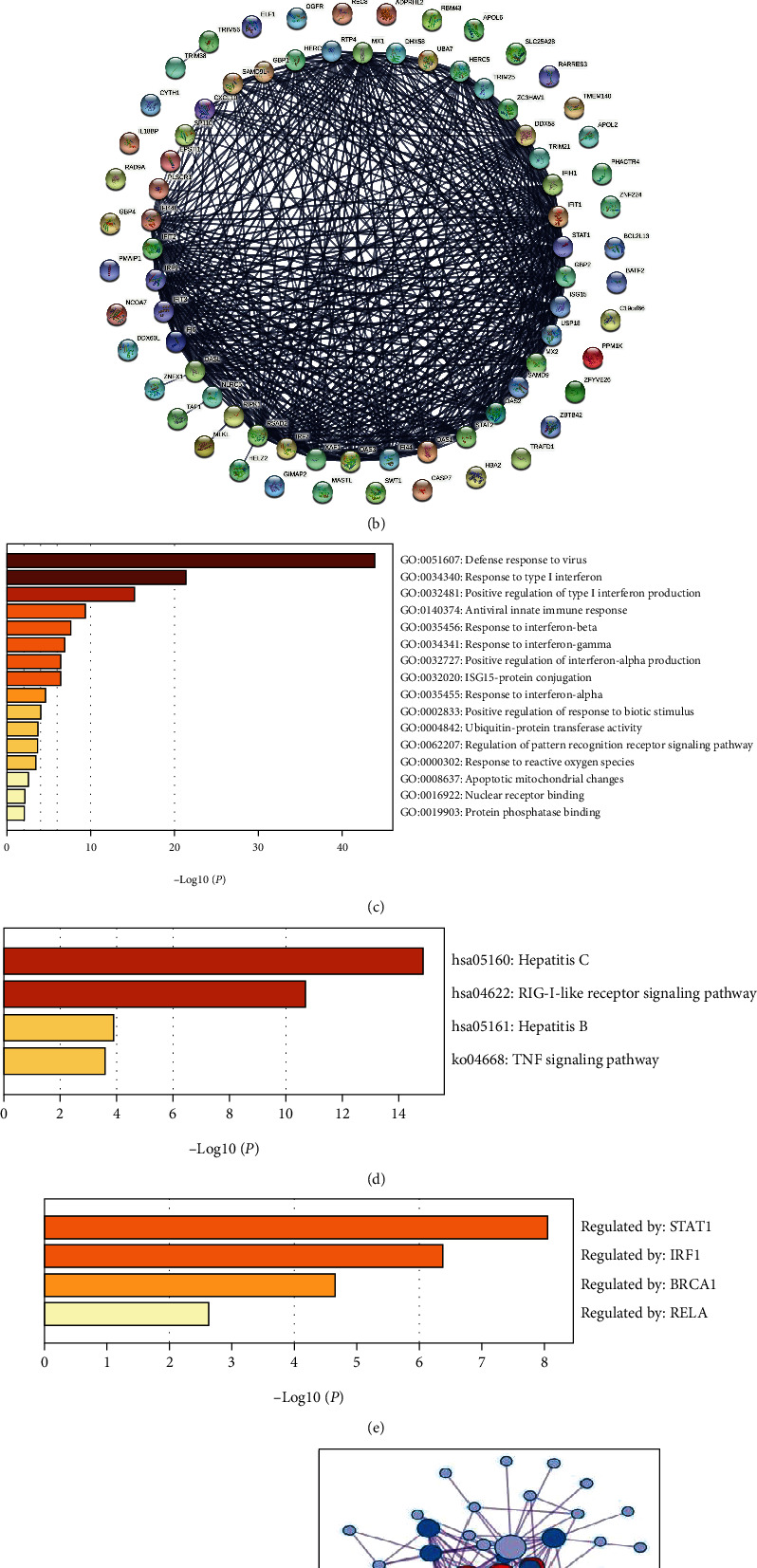
Dataset selection and DEG enrichment analysis. (a) The volcano map of the differentially expressed genes of IFN-*α*-2b acting on fibroblasts in the dataset GSE38652. (b) Based on the STRING database, the PPI interaction network diagram showing significant DEGs with an absolute value of logFc ≥ 1. (c–f) Enrichment analysis results based on Metascape. (c) GO enrichment analysis results; (d) KEGG pathway enrichment analysis; (e) TRRUST transcriptional regulatory network analysis results; (f) differential gene interaction relationship PPI network diagram and hub genes.

The volcano map shows the distribution of 28,553 expressed genes (Figure 3(a)). Among them, according to |logFC| ≥ 1 and *P* < 0.05 as the cut-off criteria, 78 DEGs were extracted, and the interaction relationship is shown in Figure 3(b). In order to obtain more biological information, we used the online database (Metascape) to perform GO and KEGG enrichment analyses. DEGs are divided into three functional groups: biological processes (BP), molecular functions (MF), and cellular components (CC). GO analysis shows that changes in biological processes are significantly enriched in defense response to virus, negative regulation of viral process, response to type I interferon, positive regulation of type I interferon production, and other immune response and transcriptional regulation. As for cellular components, DEGs are abundant in the perinuclear region of cytoplasm. Changes in molecular functions are mainly concentrated in 2′-5′-oligoadenylate synthetase activity, RNA helicase activity, nuclearoside-triphosphatase activity, ubiquitin-protein transferase activity, protein homodimerization activity, ubiquitin-like protein ligase binding, nuclear receptor binding, and protein phosphatase binding aspect (Figure 3(c)). KEGG pathway analysis showed that DEGs play a key role in hepatitis C/B, RIG-I-like receptor signaling pathway, and TNF signaling pathway (Figure 3(d)). The TRRUST database can provide information on how to regulate these interactions, and analysis suggests that DEG transcriptional regulation is mainly enriched in genes such as STAT1, IRF1, BRCA1, and RELA (Figure 3(e)). The protein-protein interaction (PPI) network results showed 22 hub genes and 125 connections (Figure 3(f)), including the STAT1 gene. Through the above analysis, we speculate that the role played by IFN-*α*-2b on fibroblasts is mainly regulated by STAT1, and P21, as a downstream molecule of STAT1, can regulate cell proliferation. Therefore, we verified the STAT1/P21 pathway in vitro.

### 3.3. IFN-*α*-2b Inhibits Fibroblast Proliferation

**Figure 4:**
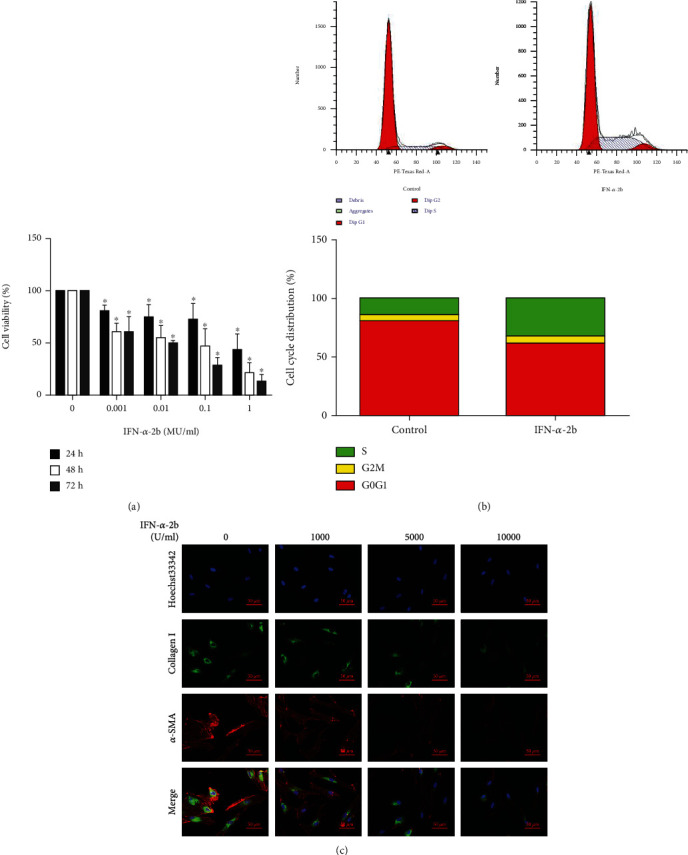
IFN-*α*-2b inhibited fibroblast proliferation and extracellular matrix secretion. (a) CCK-8 assay showed that IFN-*α*-2b inhibited fibroblast activity in a concentration- and time-dependent manner. (b) Flow cytometry analysis of cell cycle distribution showed that fibroblasts were arrested in the S phase after 48 hours of IFN-*α*-2b treatment. (c) The expression images of *α*-SMA and collagen I in cells treated with IFN-*α*-2b for 48 hours were observed under fluorescence microscope.

CCK-8 assay showed that the activity of fibroblasts decreased with the increase of IFN-*α*-2b concentration and the prolongation of treatment time. The results showed that IFN-*α*-2b inhibited the activity of fibroblasts in a time- and concentration-dependent manner (Figure 4(a)). Flow cytometry analysis showed that the proportion of IFN-*α*-2b-treated cells increased significantly in the S phase, suggesting that the cell cycle may be blocked in the S phase (Figure 4(b)). Fluorescence microscopy analysis showed that with the increase of IFN-*α*-2b concentration, the fluorescence intensity of type I collagen and *α*-SMA gradually decreased, suggesting that IFN-*α*-2b reduces the formation of extracellular matrix (Figure 4(c)).

**Figure 5:**
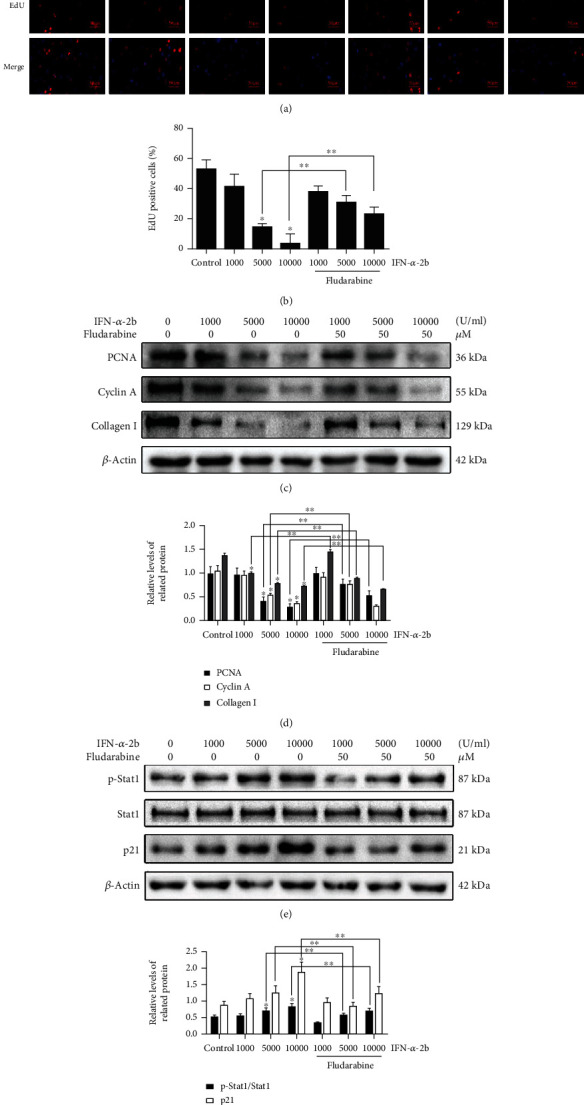
The inhibitory effect of IFN-*α*-2b on the proliferation of fibroblasts by STAT1/P21 signaling pathway. (a) After treating fibroblasts with IFN-*α*-2b or combined with fludarabine, perform EdU staining to analyze the result image. The cells shown in red are marked as positive. (b) The results of the EdU incorporation test showed that as the concentration of IFN-*α*-2b increased, the percentage of positive cells decreased significantly. This trend was partially reversed by fludarabine. (c, e) Western blotting shows the increase of PCNA, cyclin A, and collagen I and predicts the expression levels of related pathway proteins. (d, f) The analysis results showed that IFN-*α*-2b significantly reduced the relative expression levels of PCNA, cyclin A, and collagen I in a concentration-dependent manner, while increasing the relative expression levels of P-STAT1 and P21. And after fludarabine application, the trend of action was partially reversed. The histogram shows the results of three repeated detections of the gray value of the Western blot band. ^∗^Compared with the control group. ^∗∗^Compared between the two groups, *P* < 0.05 (*n* = 3).

The analysis of EdU experimental results showed that as the concentration of IFN-*α*-2b increased, EdU-positive fibroblasts decreased, the statistical results showed a significant difference, and the fludarabine group partially reversed the trend (Figures 5(a) and 5(b)). In addition, Western blot results showed that the expression levels of proliferation-related proteins PCNA, cyclin A, and type I collagen also decreased with the increase of IFN-*α*-2b concentration (Figures 5(c) and 5(d)), while STAT1 and P21 were activated with the application of IFN-*α*-2b. This is because the expression levels of p-STAT1/STAT1 and P21 gradually increased (Figures 5(e) and 5(f)). With the addition of fludarabine, the above phenomenon reversed the blocking expression trend to a certain extent.

### 3.4. IFN-*α*-2b Inhibits Fibroblast Migration

**Figure 6:**
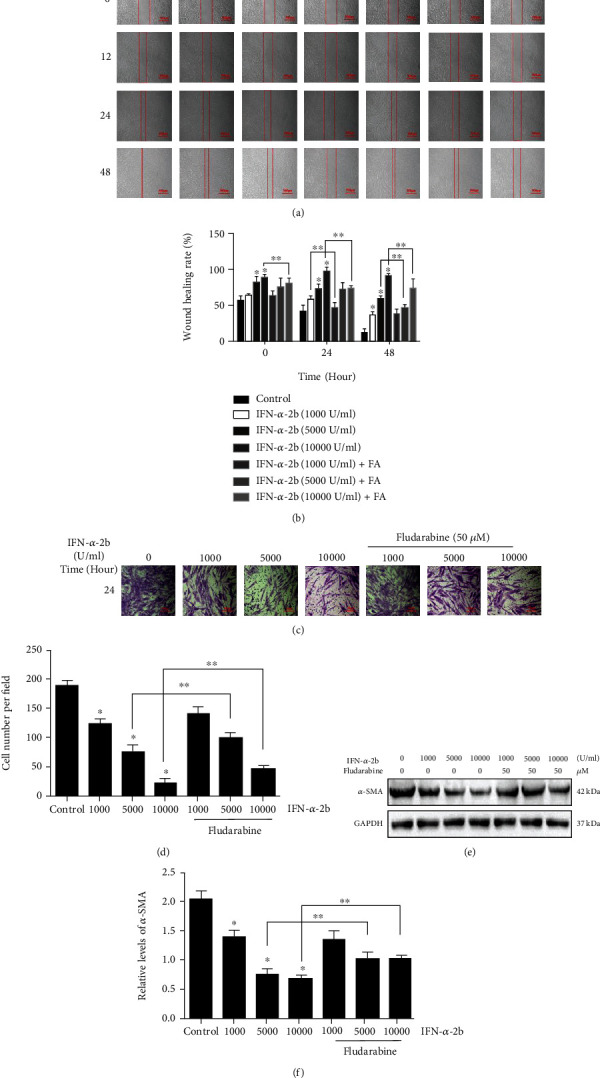
IFN-*α*-2b inhibits the migration of fibroblasts by STAT1/P21 signaling pathway. (a, b) In vitro wound healing experiments showed that the IFN-*α*-2b treatment group significantly inhibited the migration rate of fibroblasts. (c, d) Compared with the control group, the number of cells passing through the cell bottom membrane in the IFN-*α*-2b treatment group was significantly reduced. And it is concentration-dependent. (e, f) Western blotting shows that the IFN-*α*-2b treatment group can reduce the relative expression level of *α*-SMA, while the fludarabine and IFN-*α*-2b coapplication groups can partially reverse the above-mentioned inhibitory trend of IFN-*α*-2b. Each sample randomly selects 10 microscope fields and conducts 3 independent experiments. ^∗^Compared with the control group. ^∗∗^Compared between the two groups, *P* < 0.05 (*n* = 3).

After treatment with IFN-*α*-2b, the migration of fibroblasts was inhibited. Compared with the control group, the wound healing rate of the IFN-*α*-2b group decreased in a dose-dependent manner (Figures 6(a) and 6(b)). The results of Transwell migration experiment showed that the concentration of IFN-*α*-2b increased. As the concentration of IFN-*α*-2b increases, the number of cells on the bottom membrane of the migration chamber gradually decreases (Figures 6(c) and 6(d)). Western blot showed that the expression level of *α*-SMA in the IFN-*α*-2b application group showed a downward trend, and the fludarabine application group also saw a partial reversal of the inhibition trend (Figures 6(e) and 6(f)). In summary, the above experimental results show that IFN-*α*-2b can inhibit the proliferation and migration of fibroblasts, and the STAT1/P21 signaling pathway may be involved in the regulation.

### 3.5. Crosstalk between STAT1/P21 and TGF*β*/Smad Signaling Pathways

**Figure 7:**
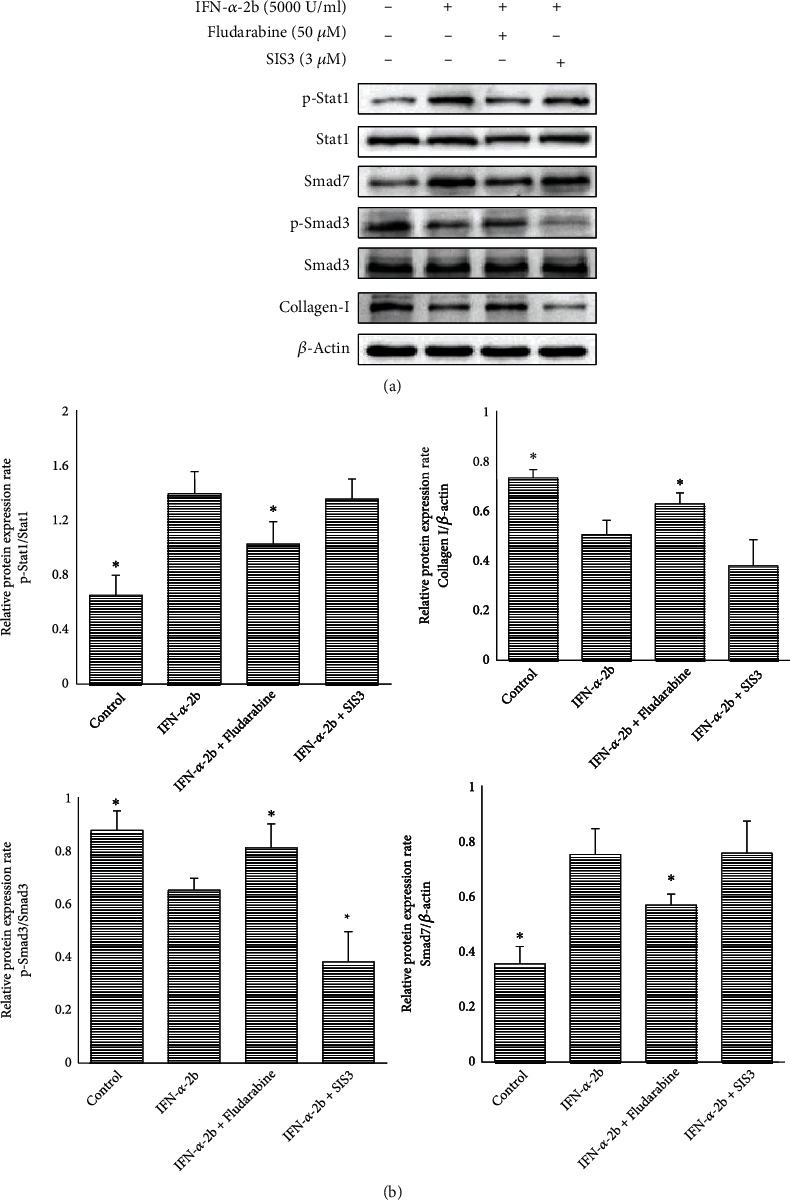
Crosstalk between STAT1/P21 and TGF*β*/Smad signaling pathways. (a) Western blotting showed the expression levels of p-Stat1, Smad7, p-Smad3, and collagen I in different groups. (b) The analysis results showed that IFN-*α*-2b significantly reduced the relative expression levels of p-Smad3 and collagen I, while increasing the relative expression levels of p-STAT1 and Smad7. Within the fludarabine prestimulation group, the effect trend was partially reversed. Compared with the IFN-*α*-2b group, the change trend of p-Smad3 and collagen I was enhanced in the SIS3 prestimulation group, but only the former had statistical significance, while p-STAT1 and Smad7 had no significant change. The histogram shows the results of three repeated detections of the gray value of the Western blot band. ^∗^Compared with the IFN-*α*-2b group, *P* < 0.05 (*n* = 3).

Our team before published an article on IFN-*α*-2b treatment of epidural postoperative adhesion, which showed that IFN-*α*-2b can play a role by inhibiting TGF*β*/Smad signal pathway [17]. The relevant signaling pathways involved in this manuscript are experimentally verified by us after screening the network database. The results showed that IFN-*α*-2b could inhibit proliferation and fibrosis after knee surgery through STAT1/P21 pathway. For these two signaling pathways in the study of the relationship, we conducted the experiment verification. Here, we added Smad3-specific inhibitor (SIS3) and STAT1-specific inhibitor fludarabine for prestimulation [21, 22], and the results showed that STAT1 phosphorylation was activated by IFN-*α*-2b application and Smad7 expression was increased (which inhibited Smad3 phosphorylation). In addition, Smad3 phosphorylation and type I collagen expression were significantly decreased, which could be reversed by fludarabine pretreatment. The activation of p-STAT1 and the expression of Samd7 decreased, the phosphorylation of Smad3 increased, and the expression of type I collagen increased. However, the phosphorylation level of Smad3 was significantly reduced in the group treated with SIS3 in advance, although the expression of type 1 collagen also showed a downward trend, but there was no statistical difference, and the activated expression levels of Smad7 and p-STAT1 did not change significantly (Figures 7(a) and 7(b)). According to the experimental results, we inferred that there is crosstalk between STAT1/P21 and TGF*β*/Smad signaling pathways, which may be due to the phosphorylation and activation of STAT1, which stimulates the increase of Smad7 and then plays a role in inhibiting the activation of Smad3.

## 4. Discussion

As mentioned earlier, excessive proliferation of fibroblasts and excessive secretion of extracellular matrix (ECM) in the surgical area can lead to fibrosis [10], while collagen is one of the important components of extracellular matrix, which can promote cell proliferation and migration [23]. Fibroblasts are transformed into myofibroblasts during fibrosis, resulting in excessive synthesis and deposition of extracellular matrix proteins [24, 25]. The expression of *α*-SMA is considered to be a specific marker of myofibroblasts [26]. Clinical studies have reported that the expression of *α*-SMA increased significantly in joint fibrosis specimens [27], and the high expression of *α*-SMA is also closely related to the proliferation and migration of fibroblasts [28, 29]. In this study, both Masson's staining and Sirius red staining showed a decrease in collagen deposition in the IFN-*α*-2b treatment group. The results of in vitro experiments also suggested that the expression of collagen I and *α*-SMA decreased in the IFN-*α*-2b-treated group.

A number of studies have reported that IFN-*α*-2b can activate STAT1, resulting in the phosphorylation of STAT1 [30, 31]. STAT1 is a member of STAT protein family. In response to cytokines and growth factors, STAT family members are phosphorylated by receptor-related kinases and then form homodimers or heterodimers, which are transferred to the nucleus as transcriptional activators. It has been reported that the mice, struck off the STAT1 gene, are more likely to have chemically induced lung and liver fibrosis [32]. In the skin model, the expression of collagen and *α*-SMA in the granulation tissue around the wound of mice with fibroblast STAT1 gene defects increased, and the perivascular fibrosis increased significantly. These results indicated that STAT1 plays a role in tissue repair [33]. As a key element of IFN-*α*-2b signal transduction, the function of STAT1 is mainly determined by its phosphorylation state. As an active form of STAT1, p-STAT1 has been shown to inhibit tumor growth by regulating cell cycles [34]. As a regulator downstream of STAT1, p21 is the first identified cyclin-dependent kinase inhibitor (CKI) protein member. P21 can bind to several compounds of cyclins and cyclin-dependent kinases (CDK), such as cyclin A/CDK2, cyclin E/CDK2, cyclin D1/CDK4, and cyclin D2/CDK4 [35, 36]. The increased expression of p21 can reduce the expression of cyclin, thus inducing cell cycle stagnation and inhibiting cell proliferation [37], which is consistent with our experimental results. In this study, with the increase of IFN-*α*-2b concentration, the expression of p-stat1 and p21 increased, while cell proliferation and migration were inhibited. The results of the pretreatment group with STAT1-specific inhibitor (fludarabine) partially reversed this trend. This means that STAT1/p21signaling pathway is involved in the antiproliferation and migration inhibition of IFN-*α*-2b. In this study, we explored the relationship between STAT1/P21 and TGF*β*/Smad signaling pathways. After all, the TGF*β*/Smad signaling pathway plays a crucial role in the formation of fibrosis. The results suggest that there is crosstalk between the two signal pathways; that is, the activation of Stat1 can promote the increase of the expression of Smad7, thus preventing the expression of Smad3, which is similar to some previous studies [38, 39].

During our experiment, there were no delayed wound healing, epidermal necrosis, surgical incision infection, and death. However, we did not explore the application of larger dose and time, nor did we screen the optimal application concentration of drugs, which is also the deficiency of this experiment. In addition, the formation mechanism of fibrosis is very complex. In addition to the proliferation and migration of fibroblasts, inflammatory response is also involved in the formation of arthrofibrosis [40]. Interestingly, STAT1 acts as a transcription factor in regulating proinflammatory and anti-inflammatory responses, making STAT1 an attractive anti-inflammatory target [41]. However, these were not measured in this study. We will continue to refine these explorations in future research. We are looking forward to more comprehensive and in-depth research and application.

## 5. Conclusion

In conclusion, this study firstly verified that IFN-*α*-2b can reduce surgery-induced arthrofibrosis by inhibiting fibroblast proliferation and migration, which may be related to the regulation of STAT1/p21 signaling pathway. Our study provides a new therapeutic target for intervention in arthrofibrosis.

## Data Availability

The datasets supporting the conclusions of this article are included within the article.

## Abbreviations

STAT1: Signal transducer and activator of transcription 1
*α*-SMA: *α*-Smooth muscle actin
ECM: Extracellular matrix
PCNA: Proliferating cell nuclear antigen
DEGs: Differentially expressed genes
IFN-*α*-2b: Interferon-alpha-2b
PPI: Protein-protein interaction
HE: Hematoxylin-eosin.
